# Supplementary material for: Behavioral Nudges to Encourage Appropriate Antimicrobial Use Among Health Professionals in Uganda
Source: Antibiotics (Basel). 2024 Oct 29;13(11):1016. doi: 10.3390/antibiotics13111016 (PMC11591260; doi:10.3390/antibiotics13111016)
Supplement: Supplementary file 1 [file antibiotics-13-01016-s001.zip › S3_Sample Intervention Timeline.pdf]

S3: Sample Intervention Timeline

Sunday	Monday	Tuesday	Wednesday	Thursday	Friday	Saturday
Week 1	Send Perceived Monitoring Letter					
	**Collect Leaderboard #1 Data**					
Week 2						
	**Collect Leaderboard #1 Data**					
Week 3			Post Ward Leaderboard #1			
	**Collect Leaderboard #2 Data**					
Week 4		Hold Educational Workshop #1				
	**Collect Leaderboard #2 Data**					
Week 5			Post Ward Leaderboard #2			
Week 6		Hold Educational Workshop #2				

1 | Copyright © 2023 For information contact Deloitte Global.
