# Supplementary material for: Behavioral Nudges to Encourage Appropriate Antimicrobial Use Among Health Professionals in Uganda
Source: Antibiotics (Basel). 2024 Oct 29;13(11):1016. doi: 10.3390/antibiotics13111016 (PMC11591260; doi:10.3390/antibiotics13111016)
Supplement: Supplementary file 1 [file antibiotics-13-01016-s001.zip › S1_Round 1 Interview Guide.pdf]

S1: Round 1 Interview Guide

Round 1 KI Interviews: Interview Guide

This will be administered to 10-20 key informants, including high ranking hospital officials and health care providers in the five MTaPS-supported study hospitals, as well as members of the national AMS Technical Working Group in Uganda.

**MEETING DETAILS: Formative Research, Understanding the Process and Context Surrounding the Prescribing of Antibiotics in Uganda**			
Date:		Time:	
Zoom Meeting Information:		Key Informant & Job Title (Role):	
**FACILITATION TEAM**			
Facilitator:		Note-Taker:	

INTRODUCTIONS

Introduce yourself and thank the interviewee for taking the time to meet with the team:

- **[Interviewer]**: Dear [Name], thank you for taking the time to meet with us. Before we begin, we would like to briefly introduce ourselves. My name is [Interviewer Name] and I am a member of [institution]. I will be the lead interviewer today, and would like my colleague to introduce themself.
- **[Note taker]**: Hello, my name is [Name]. I am also a member of [institution] and I will help facilitate and capture notes.

PROVIDING CONTEXT

- The USAID Medicines, Technologies, and Pharmaceutical Services (MTaPS) Program understands that antibiotic resistance is an area of growing concern for Uganda's Ministry of Health. Based on these concerns, MTaPS with its core partner Deloitte is interested in exploring approaches to support the appropriate use of antibiotics and adherence to prescribing guidelines among health care professionals in Uganda.
- The purpose of this interview is to better understand your perspectives on the social norms, hospital processes, and health system policies that influence antibiotic prescribing in hospital settings.
- We are conducting approximately 10 interviews with expert informants such as yourself.
- Have you had the opportunity to review the consent form? We will briefly cover a few key points.
- Our conversation will be no more than 30 minutes and is completely confidential. Individual responses will not be directly attributed to you – only the themes across the interviews will be aggregated and summarized in the final report.
- You may withdraw from the interview at any time, and we will delete all information collected up to that point. You may also choose not to answer specific questions during the interview. It is completely fine and there will be no negative impacts if you would like to withdraw or choose not to answer a given question.
- We would like to record this session if it is ok with you.

- Recordings will be maintained by the Deloitte team on our secure VPN protected network and accessed on password protected computers and folders. Only members of the research team will have access to files.
- At the conclusion of the study, the research team will submit data that is not linked to any key informant to USAID Bureau for Democracy Development & Innovation (DDI). All data shared with USAID and others will be anonymous. Your answers will not be linked to your name.

- Do you have any questions about what we've talked about?
- Do you consent to participating in the interview? Do you consent to being recorded?
- *(If "Yes")* We will now begin the recording. Once we start recording, we will confirm that you consent and then we will begin with the interview questions.
- *(If "No")* Thank you so much for your time.

**Interview Questions to KI (may or may not be a prescriber)**	
**Individual Factors**	
**Question 1**	Could you please describe the process, or give 1 or 2 examples, of how you or a prescriber in your facility decides if an antibiotic should be prescribed for a patient? How quickly do you or a prescriber determine whether a patient should be prescribed an antibiotic? How do you or a prescriber decide which antibiotic to prescribe? What do you or a prescriber consider? Thank you.
Notes	
**Question 2**	Please tell us how familiar you are with prescribing guidelines such as the **Uganda Clinical Guidelines**. How well do prescribers in your facility adhere to guidelines like these? On a scale of 1-5, how would you rate prescriber adherence to guidelines (with 5 being complete adherence)? If a prescriber, how do you think about them when prescribing?
Notes	
**Question 3**	Please tell us how familiar you are with prescribing guidelines such the **World Health Organization (WHO) Access, Watch, and Reserve (AWaRE)** classifications?
Notes	
**Social Factors**	
**Question 4**	How often do prescribers in your facility prescribe antibiotics? What do prescribers and administrators think and/or feel about prescribing antibiotics? (*Follow Up*: If a prescriber, what do you think about when prescribing

	antibiotics? How would you benchmark yourself compared to other prescribers at your facility, in terms of antibiotic prescription frequency?)
Notes	
**Question 5**	In your view, how do patients generally feel about being prescribed antibiotics? How much do patients generally know about antibiotics and what they are used for? Is there anything notable about how patients view antibiotics compared to other medications? How do patients discuss this with prescribers? How often do patients request antibiotics?<br>(*Follow Up:* If a prescriber, how do you respond when patients request antibiotics? Are prescribers able to see a patient’s previous history of antibiotic prescriptions?)
Notes	
**Question 6**	Are prescribers and hospital management concerned with inappropriate use of antibiotics and the problem of antimicrobial resistance (AMR)? Are they concerned with the frequency of antibiotic prescriptions? Does this seem to be more or less important than other hospital priorities?
Notes	
**Question 7**	What pressures, if any, impact prescriber decisions to prescribe antibiotics or not to prescribe antibiotics?<br>(*Follow Up:* Do you or do you think prescribers ever feel pressure to prescribe antibiotics to patients who do not need them? Do patients generally expect antibiotics to be prescribed during consultations? What factors influence how patients think about antibiotics? Are there any other pressures or factors that influence decisions to prescribe antibiotics?)
Notes	
**Environmental Factors**	
**Question 8**	Can you please describe how prescriptions are ordered by prescribers and how they are filled and administered (ex. paper vs. electronic records)? Are Rx pads formats regulated by local or national health authorities? Do they require certain fields or features? Could you please share a copy of the paper prescribing tool used?

Notes	
**Question 9**	Are health records and other health information maintained electronically or paper-based? Where is this information stored? What information is collected? Are there known data or information that are frequently missing or are there any issues collecting it?
Notes	
**Organizational Factors**	
**Question 10**	Does the hospital (or any other agency) provide any training, guidance, or oversight programs specific to antibiotic prescription or antibiotic resistance? Are these topics addressed in any ongoing education requirements? Are there other interventions related to prescriptions of any drugs going on now or that have been implemented recently?
Notes	
**Question 11**	What guidance or expectations does your hospital provide on antibiotic prescriptions? Is there a larger entity also providing guidance or policies, such as the broader health system? If so, how involved is the entity in overseeing prescription behavior and processes at this facility?
Notes	
**Question 12**	How might your hospital or health system better support appropriate antibiotic prescription among medical staff?
Notes	
**ADDITIONAL COMMENTS**	
**Question 13**	What do you think would be appropriate methods of communication for antibiotic-prescribing related messaging to prescribers? Please let us know if you have any opinions on the messaging or communication method that would be most effective.

Notes	
**Question 14**	Are there any additional comments or suggestions you would like to share with us regarding antibiotic prescribing in your healthcare setting or the Ugandan hospitals in general? Thank you.
Notes	
