# Supplementary material for: Behavioral Nudges to Encourage Appropriate Antimicrobial Use Among Health Professionals in Uganda
Source: Antibiotics (Basel). 2024 Oct 29;13(11):1016. doi: 10.3390/antibiotics13111016 (PMC11591260; doi:10.3390/antibiotics13111016)
Supplement: Supplementary file 1 [file antibiotics-13-01016-s001.zip › S2_Round 2 Interview Guide.pdf]

S2: Round 2 Interview Guide

Round 2 KI Interviews: Interview Guide

This will be administered to 7-10 key informants, including high ranking hospital officials and health care providers in the five MTaPS-supported study hospitals, as well as members of the national AMS Technical Working Group in Uganda.

**MEETING DETAILS: Formative Research, Designing an Intervention to Address Overprescribing of Antibiotics in Uganda**			
Date:		Time:	
Zoom Meeting Details:		Key Informant and Job Title (Role):	
**FACILITATION TEAM**			
Facilitator:		Note-Taker:	

INTRODUCTIONS

Introduce yourself and thank the interviewee for taking the time to meet with the team:

- **[Interviewer]**: Dear [Name], thank you for again taking the time to meet with us. Before we begin, we would like to again briefly introduce ourselves. My name is [Interviewer Name] and I am a member of [institution]. I will be the lead interviewer today, and would like my colleague to introduce themself.
- **[Note taker]**: Hello, my name is [Name]. I am also a member of [institution] and I will help facilitate and capture notes.

PROVIDING CONTEXT

- The MTaPs Team understands that antibiotic resistance is an area of growing concern for Uganda's Ministry of Health. Based on these concerns, the USAID Medicines, Technologies, and Pharmaceutical Services (MTaPS) Program is interested in exploring approaches to supporting appropriate use of antibiotics and adherence to prescribing guidelines among health care professionals in Uganda.
- The purpose of this interview is to hear your continued feedback on potential interventions to support appropriate antibiotic prescriptions and adherence to clinical guidelines. We came up with these ideas based on the feedback we received during the last round of interviews.
- Even though you provided verbal consent last time, can you please review the consent form and again state on the record that you agree to participate in this follow up round?
- Same as last time, our conversation will be no more than 30 minutes and is completely confidential. Individual responses will not be directly attributed to you – only the themes across the interviews will be aggregated and summarized in the final report.
    - You may withdraw from the interview at any time, and we will delete all information collected up to that point. You may also choose not to answer specific questions during the interview. It is completely fine and there will be no negative impacts if you would like to withdraw or choose not to answer a given question.
- We would like to record this session if it is ok with you.

- Recordings will be maintained by the Deloitte team on our secure VPN protected network and accessed on password protected computers and folders. Only members of the research team will have access to files.
- At the conclusion of the study, the research team will submit data that is not linked to any key informant to USAID Bureau for Democracy Development & Innovation (DDI). All data shared with USAID and others will be anonymous. Your answers will not be linked to your name.

- Do you have any questions about what we've talked about?
- Do you consent to participating in the interview? Do you consent to the audio recording?
  - (If "Yes") We will now begin the recording. Once we start recording, we will confirm that you consent and then we will begin with the interview questions.
  - (If "No") Thank you so much for your time.

**Interview Questions**	
**Intervention 1**	
**Question 1**	Do you think the proposed intervention that we have shared will influence prescribers to change their prescribing habits? If not, can you explain why? *If a prescriber* – how would it influence you?
Notes	
**Question 2**	Can you think of any logistical or organizational challenges that may make it difficult to implement the proposed intervention?
Notes	
**Intervention 2**	
**Question 3**	Do you think the proposed intervention that we have shared will influence prescribers to change their prescribing habits? If not, can you explain why? *If a prescriber* – how would it influence you?
Notes	
**Question 4**	Can you think of any logistical or organizational challenges that may make it difficult to implement the proposed intervention?
Notes	

ADDITIONAL COMMENTS	
**Question 5**	Please let us know if there are any additional comments you would like to share.
Notes	
